# Genome-wide identification and expression analysis of the *PsKIN* gene family in pea

**DOI:** 10.3389/fgene.2024.1510864

**Published:** 2024-12-18

**Authors:** Hao Yuan, Baoxia Liu, Guwen Zhang, Zhijuan Feng, Bin Wang, Yuanpeng Bu, Yu Xu, Yaming Gong, Zhihong Sun, Na Liu

**Affiliations:** ^1^ College of Horticulture Science, Zhejiang A & F University, Hangzhou, China; ^2^ State Key Laboratory for Managing Biotic and Chemical Threats to the Quality and Safety of Agro-Products, Ministry of Agriculture and Rural Affairs Key Laboratory of Vegetable Legumes Germplasm Enhancement and Molecular Breeding in Southern China, Institute of Vegetables, Zhejiang Academy of Agricultural Sciences, Hangzhou, China

**Keywords:** *Pisum sativum* L., PsKIN, gene expression, drought stress, salt stress

## Abstract

Peas (*Pisum sativum* L.) serve as a vital model for plant development and stress research. The *kinesin* (*KIN*) gene family, encoding essential motor proteins, remains understudied in peas. Our research conducted a comprehensive genomic analysis of the *KIN* genes in peas, identifying 105 genes categorized into seven subfamilies based on evolutionary relationships, gene structures, conserved motifs, and interaction networks. A comparative analysis with *Arabidopsis* and *soybean KIN* gene families showed a non-uniform distribution of *PsKIN* genes across subfamilies. Homology analysis revealed that the *PsKIN* family has undergone segmental duplication and is under negative selection pressures, with conserved genes on chromosomes Ps5, Ps6, and Ps7 playing a significant role in pea evolution. Transcriptomics revealed 38 *PsKIN* genes with distinct tissue-specific expression, with *PsKIN76*, *PsKIN96*, *PsKIN82*, and *PsKIN103* showing significant levels in roots, lateral roots, stems, petals, and seeds, respectively. Differential expression under drought and saline stress was observed, with *PsKIN8*, *PsKIN11*, *PsKIN54* upregulated under drought, and *PsKIN47* and *PsKIN51* under saline stress. These genes are potential candidates for improving plant stress tolerance. This study offers insights into the pea *KIN* gene family, highlighting their potential in enhancing plant stress tolerance and setting a stage for future research.

## 1 Introduction

Peas (*Pisum sativum* L.) are integral to the global agricultural and nutritional sectors. Despite their importance, the intricate nature of their genome has posed challenges for in-depth study. However, recent scientific progress has paved the way for a better understanding of their genetic diversity and its potential applications. By leveraging high-throughput genotyping methods, comprehensive genome-wide association studies have been performed, shedding light on the genetic makeup of pea germplasm resources (Aubert et al., 2022).

Peas are notable for their rich content of essential macronutrients and bioactive substances, positioning them as strong contenders for the development of functional foods with a range of health benefits (Wu et al., 2023). The unique chemical profile of pea protein isolate also presents significant opportunities for innovation in the food industry (Golovko et al., 2023). The introduction of cutting-edge genetic manipulation techniques has revolutionized pea breeding, allowing for precise genetic modifications that have a substantial impact on crop enhancement (Li et al., 2023). Despite these advancements, there are still hurdles in fully harnessing the genetic variability for nutritional traits and in the construction of genomic resources. Nonetheless, the application of omics-based strategies offers considerable promise for deciphering the genetic pathways that control nutritional attributes, while biotechnological approaches provide new avenues for enhancing the nutraceutical potential of peas. Kinesin proteins is indispensable ATP-driven microtubule motor enzymes and play a multifaceted role in cellular functions, are essential for photosynthesis in plants, and lay the foundation for further exploration of the pea gene family.

They participate in microtubule dynamics, chromosome segregation, organelle transport, and developmental processes such as cell division, expansion, and hormonal signaling (Solon et al., 2021). The interaction between *kinesins* and *KIFBP* modulates their binding to microtubules, thereby influencing a range of cellular activities (Seneviratne et al., 2023; Solon et al., 2021). Research across various plant species has identified and characterized the *kinesin* gene family, highlighting their regulatory roles, especially in early fruit development and cytoskeletal reorganization during cell division (Tian et al., 2021; Galindo-Trigo et al., 2020). The expansion of plant *kinesin* families underscores their critical role in facilitating plant-specific cytoskeletal rearrangements. The study of specific *kinesin* members, such as *AtKRP125b* in *Arabidopsis thaliana*, has provided insights into their involvement in mitosis and potential extranuclear functions, (Strauß et al., 2021). Gaining insights into the functions and regulatory mechanisms of plant *kinesins* is crucial for understanding the molecular basis of plant growth and development.

In our study, we employed the most current genomic data and search methodologies to identify 105 *PsKIN* (*Kinesin*) genes in pea. The evolutionary and structural characteristics of these *PsKINs* were elucidated through phylogenetic analysis, gene structure examination, identification of conserved domains, and interaction network predictions. Additionally, we systematically investigated the expression patterns of *PsKINs* across 20 RNA-seq samples, and a subset of hormone response candidates was further validated through transcriptome sequencing analysis. This research establishes a foundation for future functional studies of *PsKINs* and uncovers their roles in hormone response and plant growth regulation.

## 2 Materials and methods

### 2.1 Identification and sequence analysis of KIN genes from pea

The complete genomic sequence of the pea plant was sourced from the National Center for Biotechnology Information (NCBI) database. A hidden Markov model (HMM) was crafted using a variety of *kinesin* (*KIN*) protein sequences from different species, which are accessible on the NCBI (GCA_024323335.1) platform. The HMMER software package (version 3.3.2) was then applied to perform searches against the local protein database of the pea plant, with an E-value threshold set at 10^-5 to filter results (Supplementary Table S1). This process resulted in the identification of a preliminary list of *KIN* candidate genes, which was subsequently refined by eliminating any duplicates. This method has been reported in the literature (Potter et al., 2018). In *rice*, *Arabidopsis*, and *soybean*, the HMMER model construction was also used (Guo et al., 2008; Chen et al., 2014).

To substantiate the authenticity of these candidate sequences, the NCBI Conserved Domain Database (CDD) and the SMART tool were employed. These tools were used with an E-value threshold of 10^-5 to filter the sequences based on the presence of the characteristic *KIN* domain. The *KIN* genes that passed this validation were systematically renamed according to their respective chromosomal locations within the pea genome.

For the prediction of subcellular localization, the Cell-PLoc software (version 2.0) was engaged. Additionally, the ExPASy ProtParam tool was leveraged to forecast various physicochemical attributes of the proteins, encompassing molecular weight (MW), isoelectric point (pI), instability index, and grand average of hydropathicity (GRAVY).

### 2.2 Phylogenetic relationship, gene structure and conserved motifs analysis

To elucidate the phylogenetic connections among the *kinesin* (*KIN*) genes of the pea plant, we conducted multiple sequence alignments involving the identified pea *KIN* proteins and those from *Manihot esculenta*, *Populus trichocarpa*, and *A. thaliana*. The sequence alignment was executed using the ClustalW tool, and the resultant data were utilized to construct a maximum likelihood (ML) phylogenetic tree with IQ-TREE 2 software, applying a bootstrap value of 1,000 to ensure the robustness of the tree topology.

The chromosomal positions and the exon-intron structures of the *KIN* genes were extracted from the genome annotation files. These files were sourced from the Ensembl Plants database (http://plants.ensembl.org/index.html). The physical locations of the *KIN* genes on the chromosomes were visualized using the MapGene2Chromosome v2.0 (http://mg2c.iask.in/mg2c_v2.0/). The exon-intron structures were graphically represented with the aid of the Gene Structure Display Server (GSDS2.0) (http://gsds.cbi.pku.edu.cn/).

Furthermore, to identify the conserved domains within each *KIN* protein sequence, we employed the MEME tool (http://meme-suite.org/). The search for motifs was configured to identify up to 10 distinct motifs, with all other parameters left at their default settings to ensure a thorough and unbiased search for conserved elements.

### 2.3 Chromosomal distribution, syntenic analysis, and predicting the protein-protein interaction network of the PsKINs

The genomic coordinates and structural attributes of genes on the chromosomes of pea were extracted from the GFF3 file, which was sourced from the pea genome database (htKIN://www.peagdb.com/download/). Subsequently, the MapGene2Chrom web v2 (http://mg2c.iask.in/mg2c_v2.0/) was applied to graphically represent the *PsKIN* genes on their respective chromosomes. The analysis of synteny among the *KIN* genes across pea, *A. thaliana*, and *Glycine max* was conducted using the Multiple Collinearity Scan Toolkit (MCScanX) and TBtools. The genomic datasets for *A. thaliana* and *G. max* were obtained from the Phytozome12 database (htKIN://phytozome.jgi.doe.gov/pz/portal.html).

The analytical capabilities of MCScanX were leveraged to determine the Ka (non-synonymous substitution rate) and Ks (synonymous substitution rate) for gene pairs involved in both segmental and tandem duplications. The Ks values were used to calculate the dates of duplication events (T) according to the following equation: T = Ks/2λ, with λ = 1.5 × 10^−8^ s for dicots (Bian et al., 2020). The Ka/Ks value was further employed to identify the selection mode of the *PsKINs*. STRING (htKIN://string-db.org/) was used to construct the functional interaction network of the proteins.

### 2.4 Material and treatments

The propagation of the ‘Zhewan No.1’ (ZW1) TM-1 seeds of variety was initiated in a substrate composed of nutrient-enriched soil within an artificially regulated greenhouse. The conditions within the greenhouse were tailored to provide a 16 h period of light exposure at a steady temperature of 27°C, followed by an 8 h interval of darkness maintained at 22°C, with the atmospheric humidity consistently maintained between 60% and 80%. Peas seedlings of the TM-1 variety, which had reached a developmental stage characterized by the presence of three leaves and demonstrated signs of robust health and stable growth, were subsequently introduced to treatments designed to induce drought and salt stress. For the induction of salt stress, the root systems of the seedlings were treated with an irrigation solution containing a concentration of 300 mM sodium chloride (NaCl). In contrast, to mimic the effects of drought stress, a 20% solution of polyethylene glycol 6,000 (PEG6000) was applied to the root systems. Following the application of these stressors, leaf samples were harvested at several time points post-treatment: immediately post-treatment (designated as 0 h), as well as at 3 h and 24 h intervals. Upon collection, each leaf sample was rapidly immersed in liquid nitrogen to achieve rapid freezing, and then securely stored at a temperature of −80°C for the purpose of RNA extraction in subsequent experimental procedures.

### 2.5 Transcriptome sequencing

Post-treatment at 3 h and 24 h time points, the fresh pea seedling leaves from the untreated control group and those subjected to 300 mmol NaCl for both time intervals, as well as the leaves treated with PEG6000, were flash-frozen in liquid nitrogen and dispatched to Tianjin Jizhi Gene Technology Co., Ltd. For further analysis. In order to validate the uniformity across our experiments, triplicate biological samples were analyzed. RNA integrity was determined by agarose gel electrophoresis, revealing clear 28S and 18 S rRNA bands in the total RNA, with the 28 S intensity of band being roughly double that of the 18 S band. For the preparation of the RNA-seq library, a multi-step protocol was adhered to, encompassing total RNA isolation, mRNA purification, fragmentation, cDNA generation, end-repair of double-stranded cDNA, adapter attachment, and PCR amplification. Additionally, the RNA-seq dataset was subjected to Weighted Gene Co-expression Network Analysis (WGCNA) to assess and secure the precision and dependability of the transcriptome data.

The resulting raw sequencing reads underwent a stringent quality control process and were subsequently cleaned. These high-quality reads were aligned to the reference genome, accessible via the provided link, to facilitate the assembly of transcripts and the quantification of gene expression levels. The quality of the RNA-seq alignments was meticulously evaluated. The subsequent analysis of the transcriptome was performed utilizing a proprietary cloud-based platform developed by Tianjin Jizhi Gene Technology Co., Ltd., ensuring a comprehensive and in-depth examination of the data.

## 3 Result

### 3.1 Whole-genome characterization of KIN protein in Pisum sativum

**FIGURE 1:**
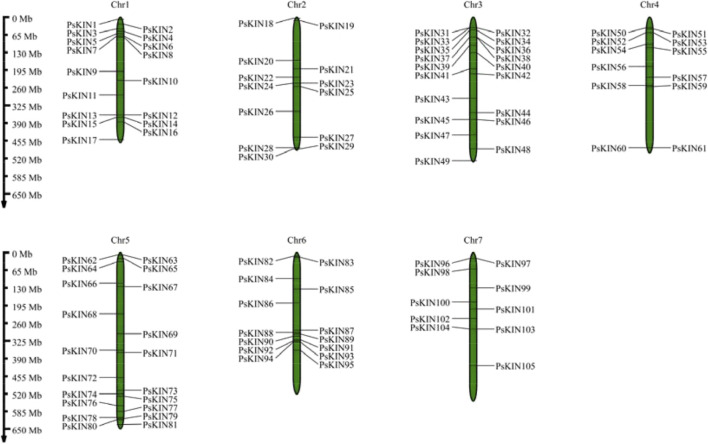
Chromosomal locations of the *PsKIN* genes on the eighteen pea chromosomes. The number of *PsKIN* genes is relatively low, and they are not distributed on every chromosome. The highest distribution of *PsKIN* genes is observed on Chr5, with seven genes present.

In our research, we initiated the investigation by employing a hidden Markov model (HMM) specific to plant *kinesin* (*KIN*) proteins, utilizing this model to conduct a comprehensive search across the entire proteome of pea. Subsequently, we proceeded to authenticate the credibility of the identified candidate *KIN* proteins. A total of 105 *KIN* proteins, characterized by the presence of a conserved *KIN* domain, were identified and designated as constituents of the pea *KIN* family, with each member named *PsKIN1* through *PsKIN105*, corresponding to their chromosomal positions (Figure 1; Supplementary Table S2).

Further analysis was conducted to elucidate the fundamental characteristics of the *PsKIN* genes, encompassing parameters such as the length of the protein in amino acids (aa), molecular weight (MW), isoelectric point (PI), instability index, and grand average of hydropathy (GRAVY). The length of the *PsKIN* proteins varied significantly, extending from 70 aa for *PsKIN60* to a substantial 2,980 aa for *PsKIN101*, with an overall mean length of 541.2 aa. The molecular weight of these proteins also exhibited a broad range, from 7.66 kD for *PsKIN60* to a notably higher value of 341.67 kD for *PsKIN101*, with an average MW of 76.50 kD. Notably, both the length and MW of *PsKIN101* exceeded those of the other *PsKIN* genes by a considerable margin. Additionally, the isoelectric points of the *PsKIN* genes spanned from 4.5 to 9.94, the instability indices ranged from 34.84 to 58.89, and the GRAVY values varied from −0.867 to 0.088.

### 3.2 Phylogenetic relationships, gene structure, conserved motif and cis-regulatory elements analysis of PsKINs

**FIGURE 2:**
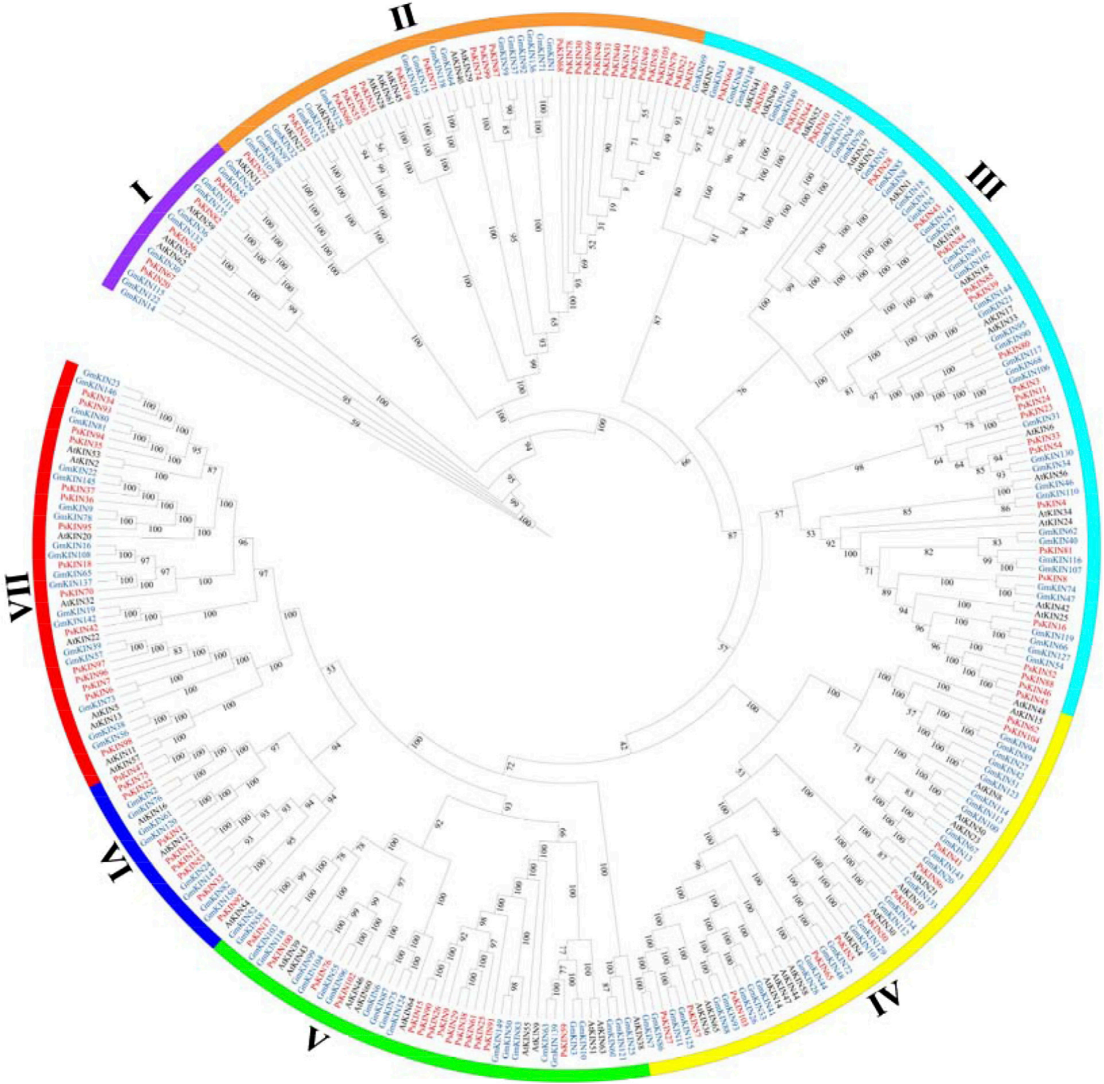
Phylogenetic tree incorporating KIN proteins from *Pisum sativum*, *Arabidopsis* and *Glycine max*. A phylogenetic tree of the *KIN* gene family was constructed by the IQ-TREE 2 software using the maximum likelihood (ML) option with 1,000 bootstrap replicates. The color of the outer ring and branches highlights different subfamilies.

To elucidate the evolutionary connections among the *KIN* gene family members in pea (comprising 105 *PsKINs*), *Arabidopsis* (encompassing 65 *AtKINs*), and *G. max* (consisting of 150 *GmKINs*), we developed a maximum likelihood (ML) phylogenetic framework. This framework was utilized to delineate the phylogenetic affinities among the species in question (Figure 2). Within the ML phylogenetic tree, the 320 *KIN* genes were categorized into seven distinct subfamilies, following the taxonomic and topological criteria established by prior research. The distribution of the 105 *PsKINs* across these subfamilies was found to be non-uniform, as detailed in Supplementary (Supplementary Table S2). Among the subfamilies, Subfamily III was identified as the most populous, housing 25 *PsKIN* genes. In contrast, Subfamily I was the least represented, with only 5 *PsKIN* genes. This differential distribution underscores the diversity and evolutionary complexity within the *KIN* gene family across the examined species.

**FIGURE 3:**
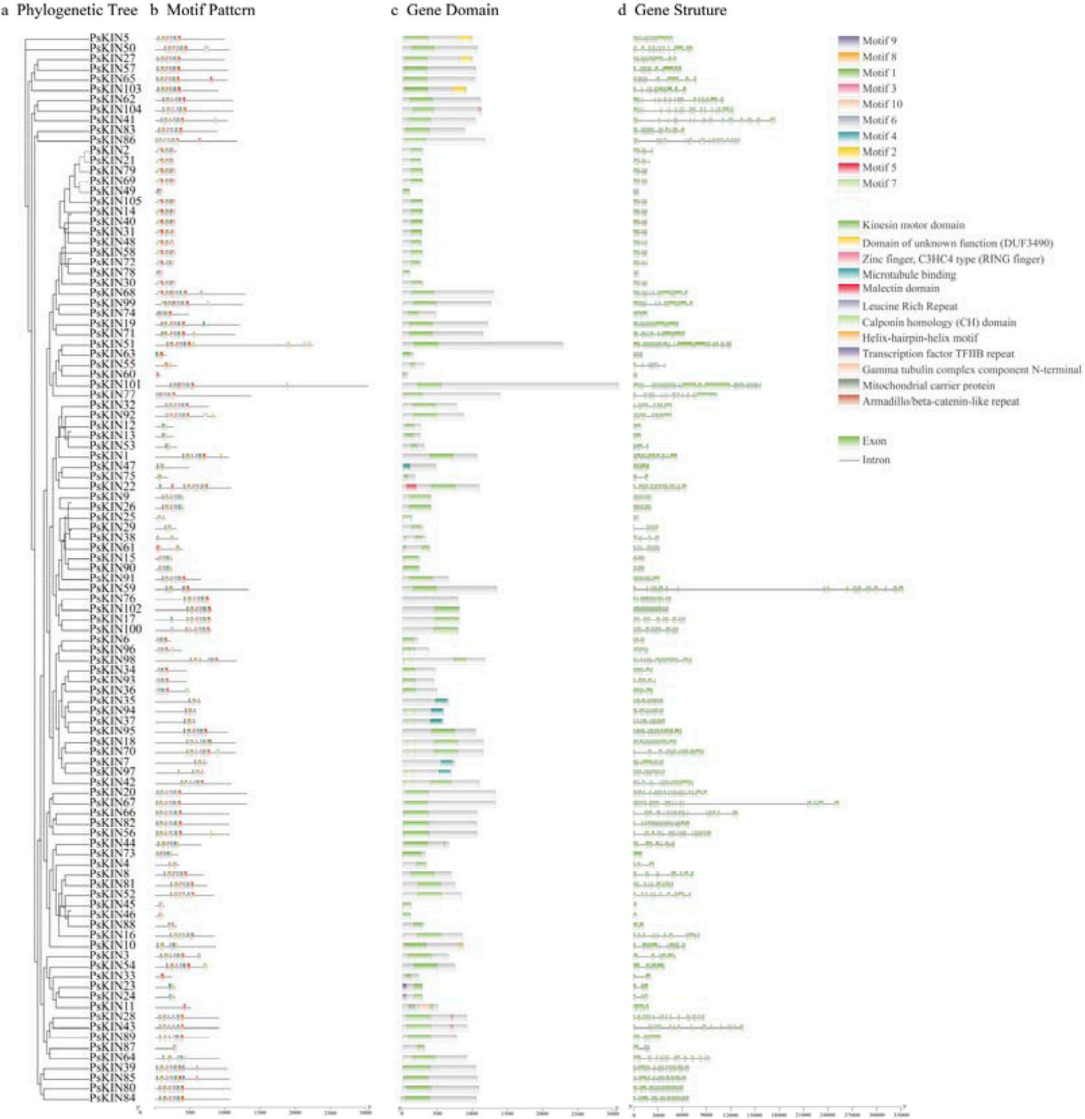
The phylogenetic relationship, conserved motifs and gene structure of *PsKINs*. **(A)** The maximum likelihood (ML) phylogenetic tree of *PsKIN* proteins was constructed using full-length sequence with 1,000 bootstrap replicates; **(B)** Distribution of conserved motifs in *PsKIN* proteins. A total of 10 motifs were predicted, and the scale bar represents 100 aa; **(C)** Distribution of *KIN* domain of *PsKINs*; **(D)** The gene structures of the *PsKINs*, include introns (black lines) and exons (green rectangles). The scale bar indicates 1,000 bp.

In our investigation, we conducted a comprehensive analysis of the gene structure and the conserved motifs present within the 105 *PsKIN* proteins, correlating these findings with their phylogenetic relationships (Figures 3A–D). Utilizing the MEME online tool, we identified 10 conserved motifs (motifs 1–10; Figure 3B) across the *PsKIN* proteins. The analysis highlighted variations in the composition of these motifs, with a general trend showing that the motif distribution aligns well with the classification into subfamilies. Notably, motifs 5, 6, and 7 emerged as the predominant motifs across all *PsKIN* genes.

Concurrently, we examined the gene domains of the *PsKINs*, presenting a visual representation of the conserved *KIN* domains mapped directly onto the genetic structure of the pea (Figure 3C). Our findings indicated the presence of 12 distinct types of *KIN* domains, with the majority of genes anticipated to encompass the “*Kinesin* motor domain”.

Further analysis of the exon count within the 105 *PsKINs*, as per the pea GFF annotation file, revealed a range from 1 to 36 exons (Figure 3D). A significant number of *PsKINs* were found to possess more than 10 coding sequences (CDSs). *PsKINs* from related subfamilies displayed a tendency towards similar exon/intron structures, indicating a strong correlation between phylogenetic relationships and gene structure within the gene family. This correlation underscores the evolutionary significance of gene structure in shaping the diversity and function of the *KIN* gene family in pea.

**FIGURE 4:**
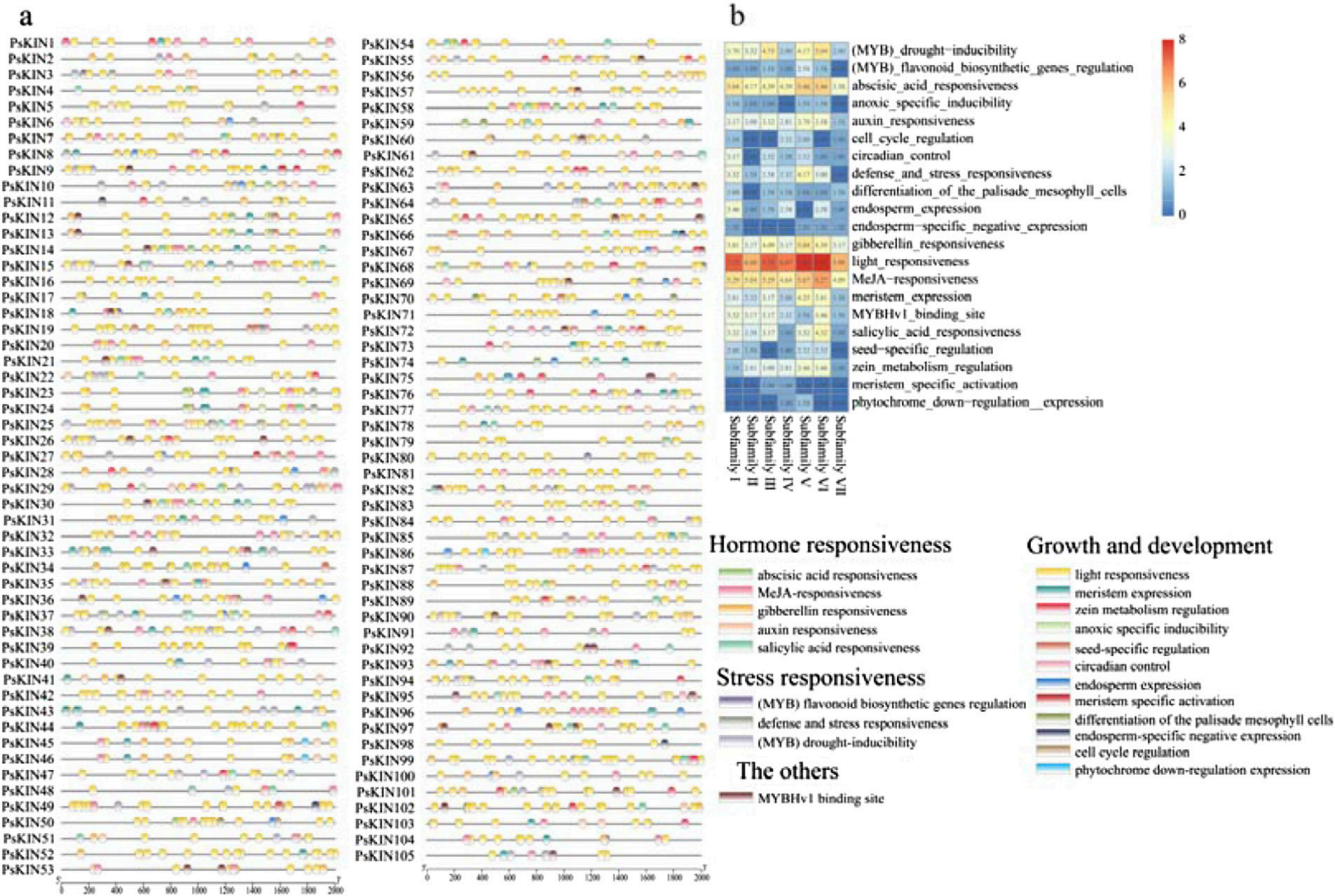
The CREs on the putative promoter of the *PsKINs*. **(A)** Distribution of CREs identified in the 2000 bp upstream promoter region of *PsKINs*; **(B)** The number of CREs on putative promoters of *PsKINs*. The numbers in the heatmap represent the quantity of elements.

Cis-regulatory elements (CREs), which are non-coding DNA sequences located within the promoter region of genes, play a pivotal role in gene expression and are integral to the regulation of a multitude of biological processes. In our study, we harnessed the 2000 base pair (bp) promoter sequences upstream of the identified *PsKIN* genes to forecast the presence of CREs, utilizing the PlantCARE database as a predictive tool. A total of 2,111 CREs sites, deemed representative, were culled from the predictive outcomes and are presented in Figure 4A. Within this set, elements associated with growth and developmental processes were found to be the most prevalent, closely trailed by those linked to hormone responsiveness, as illustrated in Figure 4B.

This analysis underscores the complexity of gene regulation within the *PsKIN* family, highlighting the significance of promoter-associated CREs in modulating gene expression in response to various developmental cues and hormonal signals. The abundance of growth and development elements, as well as hormone responsiveness elements, suggests a dynamic interplay between these regulatory factors and the expression of *PsKIN* genes, potentially influencing their roles in plant physiology and adaptation.

The CREs on the putative promoter of the *PsKINs*. (a) Distribution of CREs identified in the 2000 bp upstream promoter region of *PsKINs*; (b) The number of CREs on putative promoters of *PsKINs*. The numbers in the heatmap represent the quantity of elements.

### 3.3 Evolutionary analyses of the PsKINs within and between species

To delve into the genomic expansion mechanisms of the *PsKINs* gene family within pea, an examination of the syntenic relationships among the *PsKINs* was conducted. Out of the 105 *PsKINs*, 37 exhibited syntenic relationships, with 21 of these forming syntenic pairs that have undergone segmental duplication events within the pea genome, as detailed in Supplementary Table S4. The ratio of the rate of non-synonymous substitutions to the rate of synonymous substitutions, denoted as Ka/Ks, serves as an indicator of selective pressures acting on genes post-duplication. For the 21 segmental duplication gene pairs, the Ka/Ks ratios were found to be less than 1, suggesting that these pairs have experienced strong negative selection throughout their evolutionary trajectory.

**FIGURE 5:**
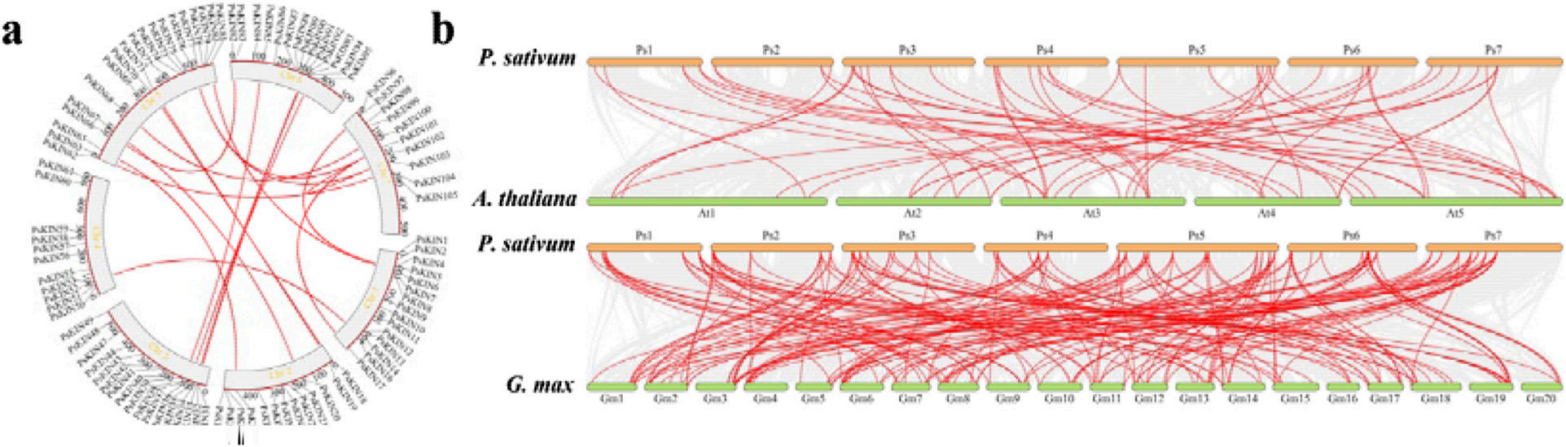
Syntenic analyses of *KIN* genes in *P. mume*, *Arabidopsis*, and *Glycine max*. **(A)** Seven chromosomes from *P. sativum* (Ps1 – Ps7) are mapped. The unit of chromosome length is Mb. Lines denote syntenic *KIN* gene pairs on the chromosomes. **(B)** The seven chromosomes of *P. sativum* (Ps1–7), five chromosomes of *Arabidopsis thaliana* (At1–5), and twenty chromosomes of *Glycine max* (Gm1–20) are mapped. The lines represent syntenic *KIN* gene pairs.

In an effort to enhance our comprehension of genetic divergence, gene duplication, and evolutionary patterns between the *KIN* gene families of *P. sativum*, *A. thaliana*, and *G. max*, syntenic relationships were scrutinized to pinpoint orthologous *KIN* genes among these species using the MCScanX tool. A total of 56 and 184 pairs of orthologous *KIN* genes were identified across three comparative analyses (*P. sativum* versus *A. thaliana*, *P. sativum* versus *G. max*), as depicted in Figure 5. It is noteworthy that certain genes exhibit multiple homologous relationships across the different species. Notably, a significant concentration of syntenic genes was observed on chromosomes *Ps*5, *Ps*6, and *Ps*7.

### 3.4 Interaction networks analysis of PsKINs

**FIGURE 6:**
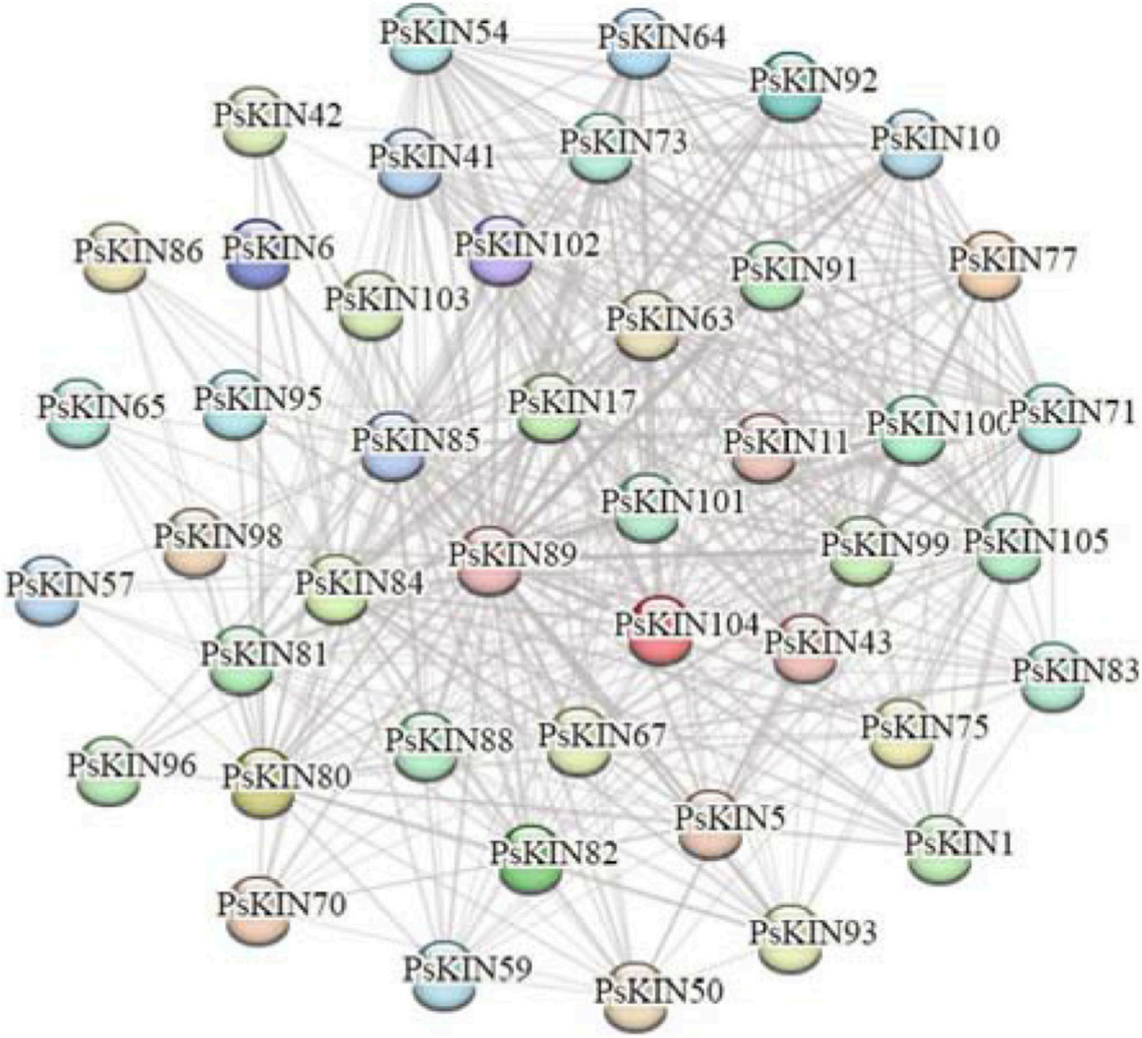
Predicted protein-protein interaction networks of *PsKINs* proteins with other proteins using STRING tool. The two circles connected by the gray line represent the interaction between the proteins.

To gain a deeper insight into the biological roles and the intricate regulatory networks associated with *PsKINs*, the potential protein-protein interactions (PPIs) among these proteins were forecasted employing an orthology-based approach. The outcomes revealed that 43 of the *PsKIN* proteins shared orthologous connections with their counterparts in *Arabidopsis*. Intriguingly, this analysis identified the presence of both physical interactions and co-expression patterns among these 43 *PsKIN* proteins (Figure 6, Supplementary Table S5). It is hypothesized that these interactions are instrumental in orchestrating plant growth and development through the modulation of protein networks.

### 3.5 Tissue-specific expression patterns of PsKIN

**FIGURE 7:**
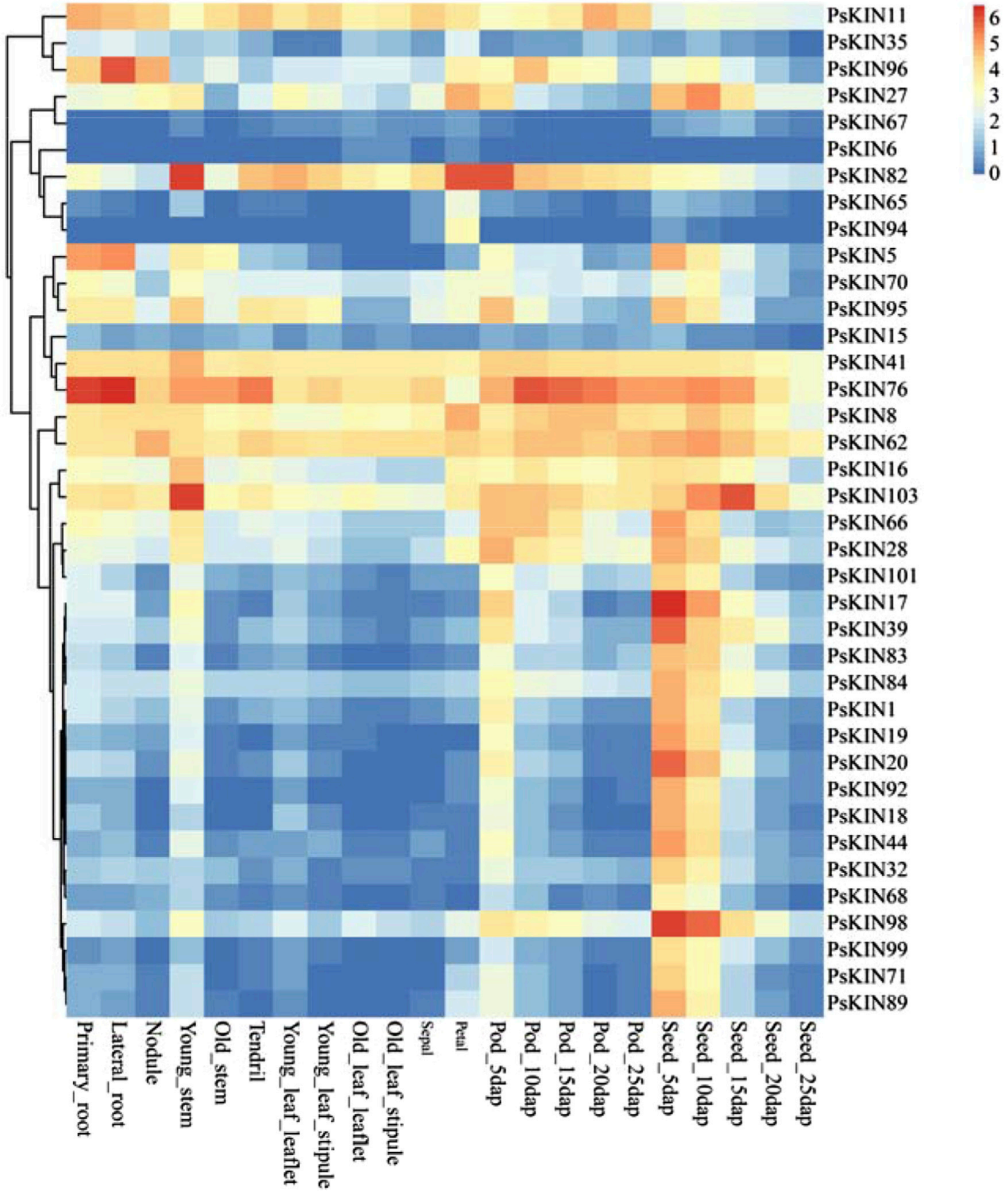
Expression profiles of the 38 *PsKIN* genes. The color scale from blue to red indicates increasing log2-transformed FPKM values.

To gain a comprehensive understanding of the expression profiles of the *PSKIN* gene family across various tissues and to anticipate their possible biological roles, an expression heatmap was developed, encompassing 38 *PSKIN* genes (Figure 7). These were meticulously picked from a collection of 105 genes, showing distinct patterns in a range of tissues including primary and lateral roots, nodules, young and mature stems, tendrils, young and mature leaves, sepals, petals, pods, and seeds.

We identified that *PSKIN76* is markedly expressed in the roots, with *PSKIN96* showing elevated levels specifically in lateral roots. Conversely, expression peaks of PSKIN82 in young stems, petals, and pods. Additionally, there is a notable upregulation of *PSKIN103* in young stems and seeds, while *PSKIN17* and *PSKIN98* are highly expressed in seeds.

Our in-depth analysis indicated that 20 genes are predominantly expressed in seeds, hinting at their involvement in seed maturation and dormancy. Meanwhile, 11 genes displayed high expression specificity in pod tissues, potentially linked to pod development and seed safeguarding mechanisms.

### 3.6 Transcriptional responses of PsKIN genes to drought and salt stress in pea

**FIGURE 8:**
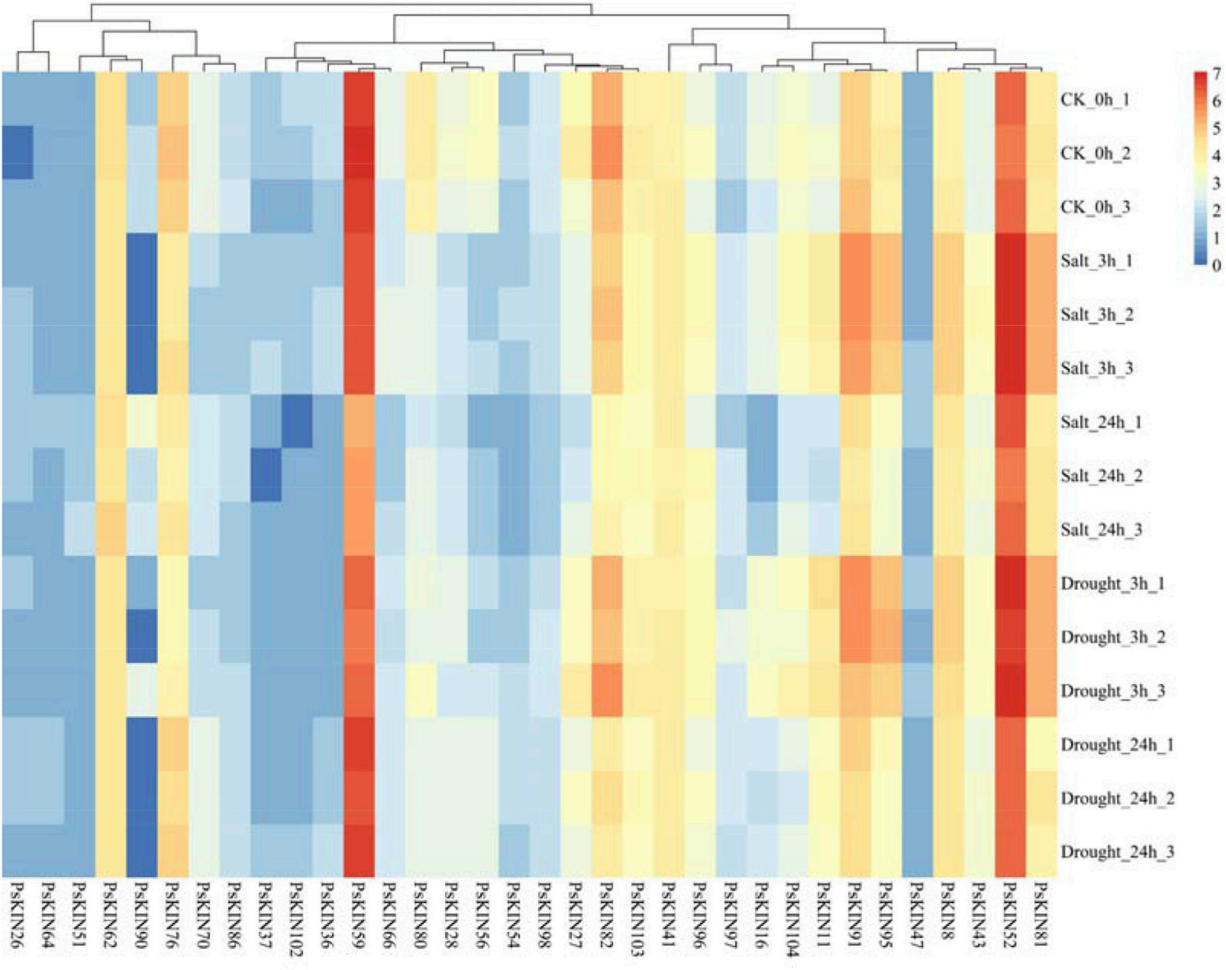
Transcriptome analysis describes the expression of 34 PsKIN genes in peas under drought stress induced by 20% PEG6000 and salt stress induced by 300 mM NaCl. Each experiment was conducted independently with a minimum of three replicates. Error bars indicate the standard deviation among replicates. “CK_0h” denotes the control group.

To explore the reaction of the KIN gene family under drought conditions, pea seedlings were placed in an environment with a 20% concentration PEG6000 solution. Leaf tissues were harvested at 3 h and 24 h intervals post-treatment for subsequent RNA extraction and transcriptome profiling. As illustrated in eight of picture, a notable fluctuation in the expression levels of 47 *PsKIN* genes was discerned, including *PsKIN8*, *PsKIN11*, *PsKIN36*, *PsKIN37*, *PsKIN54*, *PsKIN102*, and *PsKIN104*, following exposure to PEG6000 (Figure 8). This biphasic response, characterized by an initial upregulation followed by a downregulation, could be indicative of acute reaction of the plant to water scarcity and the subsequent acclimation phase. Preliminary functional analyses hint that these genes might partake in signal transduction and metabolic pathways that are instrumental in the plant’s drought stress management.

Conversely, a subset of genes, namely, *PsKIN26*, *PsKIN47*, *PsKIN54*, and *PsKIN64*, exhibited a sustained decrease in expression, potentially highlighting their involvement in long-term stress endurance mechanisms. This sustained response may be crucial for the preservation of cellular hydration and the reinforcement of antioxidant systems.

Expanding our inquiry to the realm of saline stress, seedlings were treated with escalating concentrations of NaCl, 100, 200, and 300 mM. Post-treatment analysis revealed pronounced alterations in the expression profiles of numerous *PsKIN* genes, with *PsKIN47*, *PsKIN51*, and *PsKIN90* standing out as the most significantly affected. These changes are presumed to be intimately linked to the mechanisms of the plant of acclimatization to high salinity, possibly implicating these genes in osmotic adjustment and ionic equilibrium.

## 4 Discussion

The *Kinesin* gene family in plants is tasked with the production of crucial motor proteins that are instrumental in intracellular transport and cell division. These proteins facilitate directional movement along microtubules, powered by ATP hydrolysis. The family is characterized by a functional diversity; while most *kinesins* are directed towards the plus ends of microtubules, the *kinesin-14* subclass is unique in moving towards the minus ends, thus showcasing the wide-ranging capabilities of this gene family (Yamagishi et al., 2022). In upland cotton, 159 KIN genes have been identified, which belong to the kinesin motor protein gene family and may play a crucial role in the development of cotton fibers and bolls (Zhu et al., 2024). A total of 48 KIN gene family members have been identified in watermelon, highlighting their significant role in early fruit development (Tian et al., 2021). Studies on a range of plant species, such as *A. thaliana* and *Physcomitrium patens*, underscore the evolutionary conservation and essential roles that *Kinesin* proteins play in plant biology (Herrmann et al., 2021; Yoshida et al., 2023). Phylogenetic analyses have identified around 17 distinct *Kinesin* families, each with roles varying from organelle transport to spindle pole organization, which are vital for deciphering the mechanisms of plant growth, development, and adaptation (Ali and Yang, 2020; Shen et al., 2012; Ye et al., 2022).

This investigation provides an in-depth analysis of the structural and functional diversity of the *Kinesin* (*KIN*) gene family in pea, emphasizing the interplay between conserved and divergent features across species. The study identified 105 *Kinesin* genes in the pea genome, which were classified into 7 subfamilies based on phylogenetic analysis. This classification highlights the evolutionary adaptability and complexity of the *Kinesin* gene family and its significance in various organisms. Comparative analysis of *Kinesin* gene numbers in different species—such as 48 in watermelon (Zhou et al., 2019) and 71 in moss (Wickstead andGull, 2006) —demonstrates variability in gene counts within the *KIN* family, suggesting differences in roles and classifications of these subfamilies. This variability suggests evolutionary divergence and specialization within the *KIN* gene family, reflecting the unique biological needs of each species. Further analysis of the motif composition and exon/intron structures of *PsKINs* revealed that genes within the same subfamily often share similar structural features, with a significant variation in the number of exons/introns, ranging from 1 to 36. Protein modeling results echo this pattern, showing that genes within the same subfamily have comparable secondary and 3-D structures, while those from different subfamilies exhibit diverse structural characteristics. These findings support the phylogenetic classifications and suggest that while structural features within each subfamily are conserved, the diversity among different *PsKINs* likely contributes to the functional versatility of the *KIN* gene family.

The study also explores the mechanisms behind the expansion of the *KIN* gene family in pea, identifying segmental duplicates within 21 *KIN* gene families. This indicates that gene duplication, particularly segmental duplication, plays a significant role in the evolution and expansion of the *KIN* gene family in pea. Homologous genes identified between pea and other species were predominantly traced to chromosomes *Ps*5, *Ps*6, and *Ps*7, indicating strong conservation of these chromosomes throughout pea evolution and suggesting broader implications of segmental duplications on species evolution. Such duplications are known to be instrumental in the rapid evolution of primate genes and are associated with chromosome abnormalities and genetic diseases (Lawce, 2022).

The promoter regions of *PsKIN* genes contain cis-regulatory elements essential for the regulation of plant physiological processes. These elements can be broadly categorized into those associated with plant growth and development, hormonal responses, and reactions to environmental stressors. Among these, light-responsive elements are most frequently observed, with a significant number also responding to hormones like methyl jasmonate (MeJA) and gibberellins. Light significantly impacts plant growth and development by influencing processes such as seedling establishment, leaf expansion, and photosynthesis, activating specific photoreceptors and signal transduction pathways that regulate plant physiological and developmental responses, thus modulating gene expression and plant hormone levels throughout the plant’s lifecycle (Singh et al., 2023; Yadukrishnan et al., 2021). MeJA positively affects plant growth and development by promoting seed enlargement, improving plant resistance to adversity, regulating defense mechanisms, and influencing the synthesis of metabolic products, thereby enhancing plant growth parameters and increasing its nutritional and medicinal value (Song et al., 2023; Đurić et al., 2023; Wang et al., 2022). Gibberellins affect plant performance and metabolic product composition by regulating stem elongation, germination, flowering, as well as impacting nutritional growth, sex expression, and yield (Wang et al., 2022).

Transcriptome sequencing in our study has unveiled the distinct expression profiles of 47 *PsKIN* gene family members across various plant tissues, offering critical insights into their roles in the developmental processes of plants. Notably, *PsKIN76* exhibited pronounced expression in primary and lateral roots, hinting at its potential involvement in the development of the root system. This is reminiscent of the role of DEEPER ROOTING 1 (DRO1), which is known to manipulate root growth angles and enhance rice yields under drought conditions (Uga Y et al., 2013). Drawing parallels, we hypothesize that *PsKIN76* might regulate root morphogenesis through analogous mechanisms.

The significant expression of PsKIN82 in young stems and petals raises the possibility of its contribution to the reproductive development of plants. The ectopic expression of the KNOX gene SHOOT MERISTEMLESS (STM) in *Arabidopsis* is known to induce carpel formation and promote the transformation of ovules into carpels, essential for reproductive meristem development (Scofield S et al., 2007). We anticipate that *PsKIN82* might perform a comparable regulatory function in the reproductive development of plants.

Furthermore, Elevated expression of *PsKIN103* in young stems and seeds points towards a potential role in seed development and seedling growth. The large family of receptor-like protein kinases (RLKs) participates in the regulation of plant growth and development and is capable of adapting to stress (Zhu et al., 2024). We, therefore, speculate that *PsKIN103* might serve similar functions, exerting a substantial influence on early plant development and seed maturation.

In response to abiotic stress, our study revealed that the expression of 14 *PsKIN* genes was significantly modulated, casting light on the plant’s adaptive mechanisms under harsh conditions. Under drought stress, an upregulation of genes like *PsKIN8*, *PsKIN11*, *PsKIN36*, *PsKIN37*, *PsKIN54*, *PsKIN102*, and *PsKIN104* was observed, suggesting their participation in the drought response of plant. The *GhKLCR1* gene, a kinesin light chain-related gene homologous to *AtKLCR1*, is upregulated under drought stress. Overexpression of *GhKLCR1* in transgenic *Arabidopsis* plants leads to increased sensitivity to drought stress (Li et al., 2019). *GmCAMTA12 Arabidopsis AtAnnexin2 AtWRKY1 PsKIN8*. These findings suggest that these genes may play a key role in the response of plant to drought stress.

Conversely, the downregulation of *PsKIN26*, *PsKIN47*, *PsKIN54*, and *PsKIN64* under drought stress may be linked to the drought adaptation of plant, akin to the improved drought resistance in grapevines with silenced glutathione S-transferase genes (Nerva L et al., 2022). Under salt stress, the expression changes of PsKIN47, PsKIN51, and PsKIN90 are the most significant. Transgenic rice overexpressing OsASR6 also exhibits adaptability to salt stress (Zhang Q et al., 2022). The OsTUB1 gene in rice interacts with Kinesin13A to stabilize microtubules and ion transporters, thereby conferring salt stress insensitivity to the plant (Chen et al., 2022). These findings propose that these genes might be pivotal in the plant’s response to salt stress.

In essence, our findings underscore the multifaceted roles of the *PsKIN* gene family in both plant development and stress response.

## 5 Conclusion

In our study, we identified the *PsKIN* gene family in the pea genome, consisting of 115 *PsKIN* genes that underwent in-depth characterization. This included sequence analysis, phylogenetics, assessment of gene structures and identification of conserved motifs, mapping of their chromosomal locations, analysis of their homologs, and prediction of their potential protein-protein interaction networks. To further our research, we explored the tissue-specific expression profiles of *PsKIN* genes, with particular attention to their expression dynamics under drought and salt stress conditions. The findings of this study provide profound insights and lay a solid theoretical foundation for future research aimed at uncovering the exact roles of *PsKIN* genes, especially in the context of abiotic stress.

## Data Availability

The original contributions presented in the study are included in the article/Supplementary Material, further inquiries can be directed to the corresponding authors.
